# Institutional Notes and News

**Published:** 1911-05-13

**Authors:** 


### INSTITUTIONAL NOTES AND NEWS.

**A New Institution at Southport.**

The first year's working of the new Homœopathic Cottage Hospital at Southport, Lancashire, is worth notice. The hospital received its first patient in February 1910, and has had fifty-eight successors, ten of whom occupied the two private wards. There are ten beds in the general wards. It is, therefore, somewhat larger than the average cottage hospital, and its planning and internal arrangements are interesting from the fact that they are the work of Mr. Percy Adams, the architect of the King Edward Sanatorium, Midhurst, and of Mr. Charles Holden. These buildings, however, have cost a considerable sum, which has not yet been subscribed, and the reputation for legacies which this part of Lancashire is proud of needs vindication in Fleetwood Road. The hospital, we understand, is allotted as yet no share in the public collections of Southport. There is a sliding scale of fees for patients, so elastic as to make it a contribution on their part only, but two beds are kept permanently free, chiefly for the use of dispensary patients. The hospital's supporters are proud of its up-to-dateness of construction and equipment, but, as is the case with all new institutions, it will take a little time before its revenue is equal to its deserts. Its medical officers are Dr. Lowe and Dr. Francis S. Wheeler.

**French Wit at a Hospital Banquet.**

M. Cambon, the French Ambassador, presided last Saturday at the yearly banquet of the French Hospital, which was marked by some amusing speeches. The Sisters of Charity, who undertake the nursing, came in for special praise by M. Cambon, and significance was given to the evening by the bestowal, on behalf of the French Government, of the distinctions of Chevalier de la Légion d'Honneur and of Officier d'Académie respectively upon M. Roehrich and M. Judah for their generous support of the hospital. M. Gennadius in responding to the toast of the Corps Diplomatique coupled with the Founders, said that both his occupation and the hospital's was concerned with patients, but that in his the convalescent home was represented by the pigeon hole. The Lord Mayor recalled that Sir Polydore de Keyser was the first Lord Mayor to induce the City Corporation to make a grant to the French Hospital.

**Colonel Bruce Vaughan and the Cardiff Firms.**

The energetic attempts to secure the solvency of the Cardiff Infirmary, which have been chronicled in these columns from their first success in associating its resuscitation with the National Memorial of Wales to King Edward, have met with considerable success. Now the business firms are being approached, and Colonel Bruce Vaughan, the interest of whose arguments has been often noticed, now reminds the Cardiff workmen of the lack of organised collection in the city, and of the statistical fact that if they gave a penny a week and the colliers in the interested area a shilling a year, which would make a total of £10,000 per annum, the maintenance income for the new wing would be forthcoming, and the support of the badly needed convalescent home in connection with the infirmary would be also partly gained. It is the lack or organisation, says Colonel Bruce Vaughan, which has caused the working classes of Cardiff to have given far less to their hospital than is the case in any town in England. It is hoped that the Friendly Societies and trade-unions will help Colonel Bruce-Vaughan in his attempts to remedy this lamentable admission.

**What a Hospital Dinner Can Do.**

That the Ladies' Dinner in aid of the King's College Hospital Removal Fund, which was held on Thursday last week, was a success may be judged from the fact that £59,000 was collected. Some thirty ladies presided over the tables, among whom was to have been Mrs. Asquith had not Lady Ribblesdale's death kept her away. The present position of affairs was summed up ably by Lord Selbourne, who presided. The toast was the "New King's College Hospital," and in proposing it he said that two years ago it was decided to remove the hospital from its present site on the north side of the river to Denmark Hill. The site of the new building had been criticised on the ground that it was "too far off." But surely the test of the fitness of the site did not depend on its distance from Hyde Park Corner. The only question was whether it would be able to serve a vast and a poor population. Judged by that test, he did not think a finer site could have been chosen than that which had been presented by Mr. Smith. In the new hospital there would be 600 beds, or 400 more than were available in the old one. They had met to assist in raising the last quota required for the building—£150,000. Before coming into that room they had got below £100,000, about £53,000 of the final quota of £150,000 having been collected in connection with this last great effort. Only the day before Mr. Smith was given a cheque by one gentleman for £10,000. The old site, instead of being sold and having its proceeds applied to the cost of the new building, was to be retained as part of the endowment of the new hospital. In The Hospital of March 4 (page 668) is to be found a preliminary account of the buildings.

**A Striking Improvement at Exeter.**

In accepting for the first time the post of President of the Royal Devon and Exeter Hospital, Mr. A. Tremayne Buller, at the recent quarterly meeting, after thanking the governors, said: "I accept the post with some hesitation, as I would like to have served on the committee and obtained a more detailed knowledge of the work before taking the office. But I have met with such uniform kindness from all that I am gaining more confidence." Mr. H. Langridge Lane, the honorary treasurer, then analysed the accounts of the quarter, noting especially the satisfactory increase in subscriptions, and a small additional cost in the monthly advertisements, which meant that the list of acknowledgments grew longer and that advertising paid the hospital. Mr. Lane summed up the hospital's financial position by saying that they began Lady Day 1910 with a deficit of £1,112, and that they ended the present Lady Day quarter with £597 in hand.

**New Hospital with Small Fees at Leicester.**

Gratifying progress at the Leicester Provident Dispensary was revealed at the annual meeting of the subscribers, when the Mayor, Ald. W. W. Vincent, presided. The membership now numbers 58,000. So much does the Board believe in the necessity of a hospital where members can be admitted for moderate fees that they have decided to demolish the whole of the original hospital of six beds and to erect a new hospital with modern conveniences and fittings. The contract has been recently placed at £3,889, and the Board hopes the institution will be ready for opening in a few months. Nearly one-third has already been raised towards the £6,000 required to complete and to equip the hospital. The chairman having spoken of the educative value of the institution, Sir Edward Wood referred to the great help the new hospital would be to the Leicester Infirmary, which unfortunately always had waiting cases on the list.

